# The Fragrant Power of Collective Fear

**DOI:** 10.1371/journal.pone.0123908

**Published:** 2015-05-06

**Authors:** Roa Harb, Jane R. Taulor

**Affiliations:** 1 Department of Laboratory Medicine, Yale University School of Medicine, New Haven, Connecticut, United States of America; 2 Department of Psychiatry, Division of Molecular Psychiatry, Yale University School of Medicine, New Haven, Connecticut, United States of America; Université Pierre et Marie Curie, FRANCE

## Abstract

Fear is a well-characterized biological response to threatening or stressful situations in humans and other social animals. Importantly, fearful stimuli in the natural environment are likely to be encountered concurrently by a group of animals. The modulation of fear acquisition and fear memory by a group as opposed to an individual experience, however, remains largely unknown. Here, we demonstrate a robust reduction in fear memory to an aversive event undertaken in a group despite similar fear learning between individually- and group-conditioned rats. This reduction persists outside the group confines, appears to be a direct outcome of group cognizance and is counteracted by loss of olfactory signaling among the group members. These results show that a group experience of fear can be protective and suggest that distinct neural pathways from those classically studied in individuals modulate collective fear memories.

## Introduction

Fear is an essential coping strategy under stress; however, exaggerated and persistent fear responses may contribute to the development or constitute the symptoms of anxiety/stress disorders, notably posttraumatic stress disorder (PTSD), with crippling consequences [1,2]. The classical fear conditioning model has been an immensely valuable tool for neurobehavioral studies of learning, memory, and neural plasticity as well as pathological fear [3–7]. In this paradigm, an animal is exposed to a neutral conditioned stimulus (CS) which is paired with an aversive unconditioned stimulus (US) leading to the formation of a long-lasting memory where subsequent presentation of the CS alone elicits robust fear expression [8]. Critically, humans and other social animals often experience dangerous or traumatic situations in a group. However, the impact of a collective fearful experience on subsequent memory expression, and whether the underlying neural circuitry and cellular, synaptic and molecular mechanisms significantly differ from those established with individual fearful exposures remain largely unknown. This knowledge is clinically relevant and can shed light on variability of PTSD prevalence, severity, or resilience in trauma victims [9,10].

## Materials and Methods

### Animals

Male Sprague-Dawley rats, aged 7 weeks upon arrival, were supplied by Charles River (Portage, ME, motivation USA). The rats were housed in triads under constant cage temperature (20–21°C), humidity (40–50%), and a controlled 12/12h light-dark cycle. The rats were allowed 3 weeks to acclimate to the housing environment and had free access to food and water *ad libitum*. Cage mates were always assigned to the same experimental condition. The experiments in the present study were approved by the Yale University Animal Care and Use Committee (AWA# A3230-01) and followed the NIH Guide for the Care and Use of Laboratory Animals.

### Drugs

Zinc sulfate monohydrate (Sigma, USA) was dissolved in a sterile 0.9% sodium chloride solution to a final concentration of 5% and was sprayed intranasal as 0.1 cc solution with 0.4 cc air. Ketamine and Xylazine were diluted to a final concentration of 75 mg/ml and 5 mg/ml in a sterile 0.9% sodium chloride solution and injected intramuscular at 75 mg/kg and 5 mg/kg, respectively.

### Fear conditioning

Ten week old rats were trained and tested in sound-insulated fear conditioning chambers equipped with a speaker in the side wall and a metal stainless-steel rod flooring connected to a shock generator (VideoFreeze System, Med Associates). Video images of the behavioral sessions were recorded at a frame rate of 30 frames per second via a progressive scan CCD video camera contained within each chamber and connected to a computer. Both freezing scores and motion indices were derived in real time from the video stream by computer software (Video Freeze; SOF-843). Two contexts “A” and “B” were used throughout the study. Conditioning context “A” had stainless steel rod flooring, was lit with both white and infra-red lights, and was cleaned and wiped with 70% ethanol between animals. Testing context “B” had white plastic flooring, was lit with infra-red light only (invisible to rats) and was cleaned and wiped with 1% acetic acid solution between animals. Rats were allowed to habituate for 10 minutes in conditioning context “A” either alone (Individual) or with their cagemates (Group). A custom-made transparent Plexiglas insert was then placed in the chamber and rats were randomly assigned to one of three compartments. Individual rats were randomly placed in left, center or right compartment. Group rats could still recognize the presence of their cagemates but were not in physical contact during the actual shock which could interfere with the level of current delivered. A 30-second tone (90 dBA, 5 kHz, “CS”) was then paired to and co-terminated with a 2-second scrambled foot-shock (0.8 mA, “US”). Rats received one or three CS-US pairings (See Experimental Design), separated with a 180 min inter-trial interval (ITI) and were allowed to rest for 1 min before they were returned to their home cages. Twenty-four hours later, all rats were individually or group exposed to a pre-CS baseline period of 180 min followed by three non-reinforced 30-second CS presentations with an ITI of 180 min in testing context “B” to test for long-term memory (LTM). A short-term memory test (STM) conducted in one cohort was identical with the following exceptions: testing occurred 3.5 hours after conditioning and consisted of a single non-reinforced CS presentation.

### Automated scoring

Automated scoring using VideoFreeze software has been extensively validated previously [11]. Briefly, freezing is scored based on two parameters: a motion index threshold below which motion is restricted to respiratory movements and a minimum freeze duration for the animal to be considered freezing. These two parameters were determined from best fit analysis against human observer scoring and were as follows: 18 for motion index threshold and 1 second (30 frames) for minimum freeze duration. We have separately validated both parameters against our own manual scoring (S2 Fig). Percent freezing is calculated as the time spent freezing during the CS delivery. The motion index, based on pixel differences between the digital video stream and a reference video sample of the empty chamber, can also be used to determine gross motor reactivity to shock or what is termed the unconditioned response. This motion index is a good approximation of true animal speed and can be used as a surrogate for the animal’s physical experience of the shock [11]. Average motion indices were generated for each rat during the two seconds of foot shock delivery.

### Test for anosmia

Rats were given each a quarter of a chocolate-covered peanut butter cup (Reese’s Peanut Butter Cups Miniatures) in their home cages to reduce neophobia. Twenty-four hours later, the rats were subjected to a buried food test (modified from Masini et al.[12]) in a novel test room. Each rat was placed in a new standard cage containing approximately 6 cm deep clean bedding in which a quarter of the Reese’s cup was buried in a randomly chosen corner (same corner for all rats). A stopwatch was started and the latency to find the buried food was recorded by an observer. An animal was considered to have found the food when it started eating it, usually holding it with its forepaws. The rat was returned to its home cage upon finding the food or when 10 minutes had elapsed. Twenty-four hours later, the rats underwent zinc-sulfate induced peripheral anosmia or sham treatment and the test was repeated the following day as described. The buried food, however, was placed in a new position to control for possible memory of the previous location.

### Zinc sulfate induced peripheral anosmia

Rats were randomly assigned to receive zinc sulfate induced peripheral anosmia or sham treatment; however, all three rats in the same home cage were subjected to the same assignment. Seventy-two hours following the buried food test, rats were briefly anesthetized with isoflurane and placed upside down on a custom-made inclined surface at an angle of approximately 20 degrees. Two 20-gauge hypodermic needles were modified by removing the sharp tips and flattening the openings into narrow slits. One needle was attached to a syringe with 0.1 cc 5% zinc sulfate solution and 0.4 cc air and the other was attached to a syringe with 0.1 cc saline and 0.4 cc air. Depending on treatment assignment, one needle was inserted into each nostril no deeper than 10 mm and contents quickly sprayed. The rat remained suspended until awakening for drainage to occur. All rats received treatment in the right nostril first and two hours later in the left nostril to minimize aversive effects.

### Experimental design

#### Cue-elicited fear LTM test

Three weeks following acclimatization in the colony, rats were randomly assigned to Individuals or Groups. A “weak” training cohort was fear-conditioned as described above with 1 CS-US presentation and, twenty-four hours later, individually tested for long-term memory of fear with 3 CS presentations. A “strong” training cohort was fear-conditioned with 3 CS-US presentations, and twenty-four hours later, individually tested for long-term memory of fear with 3 CS presentations.

#### Cue-elicited fear STM test

In a separate cohort, rats were randomly assigned to Individuals or Groups and fear-conditioned with 1 CS-US presentation as described above. Approximately 3.5 hours later, the rats were individually tested for short-term memory of fear with a single CS presentation.

#### Reaction to shock measurement

In a separate cohort, the following set-up was designed to determine the reaction to shock experienced by each rat. Since the automated system can accurately and reliably measure rat motion during shock delivery [11], it is necessary to limit movement inside the chamber to one rat even as all three receive the foot-shock. Fear conditioning with 1 CS-US presentation was performed as described above with the following modifications: 2 out of the 3 Group rats were anesthetized and placed on platforms inside 2 compartments such that only their feet touched the grid bars while the third rat was placed awake in the third central compartment. Each individual rat was also placed in the central compartment to mimic Group conditions. This allowed the generation of a single average motion index per chamber for both Individual and Group rats. Twenty-four hours later, Individual and “awake” Group rats were individually tested for long-term memory of fear with 3 CS presentations.

#### Cue-elicited fear testing in a Group

In a separate cohort, Individual and Group rats were fear-conditioned with 1 CS-US presentation as described above. Twenty-four hours later, they were tested for long-term memory of fear in a group with their cage mates. Manual scoring of freezing behavior was performed in both Individual and Group rats in this cohort.

#### Cue elicited fear in anosmia

In a separate cohort, rats underwent the buried food test and were randomly assigned to Anosmia or Sham condition. On day 4, zinc sulfate induced peripheral anosmia or sham procedures were performed on the rats. On day 5, the rats were subjected again to the buried food test. Pre-determined criteria for exclusion included the following: sham rats who took *longer* than and anosmic rats who took *less* than 10 minutes to find the buried food. These rats completed the study to maintain housing and test conditions as previous experiments but were excluded from the analysis. Finally, on day 6, the rats were randomly assigned to Individuals or Groups, fear-conditioned with 1 CS-US presentation, and tested twenty-four hours later for long-term memory of fear. Following data analysis, three sham rats (2 Individual, 1 Group) were excluded for >10 min latencies to find the buried food on test day.

### Statistical analysis

The data were evaluated using GraphPad Prism’s two-way analysis of variance (ANOVA) for repeated measures where appropriate. Post-hoc analysis was performed using Sidak’s or Tukey’s multiple comparisons tests where appropriate. A probability value (*p*) equal to or less than 0.05 was considered statistically significant.

## Results

**Fig 1:**
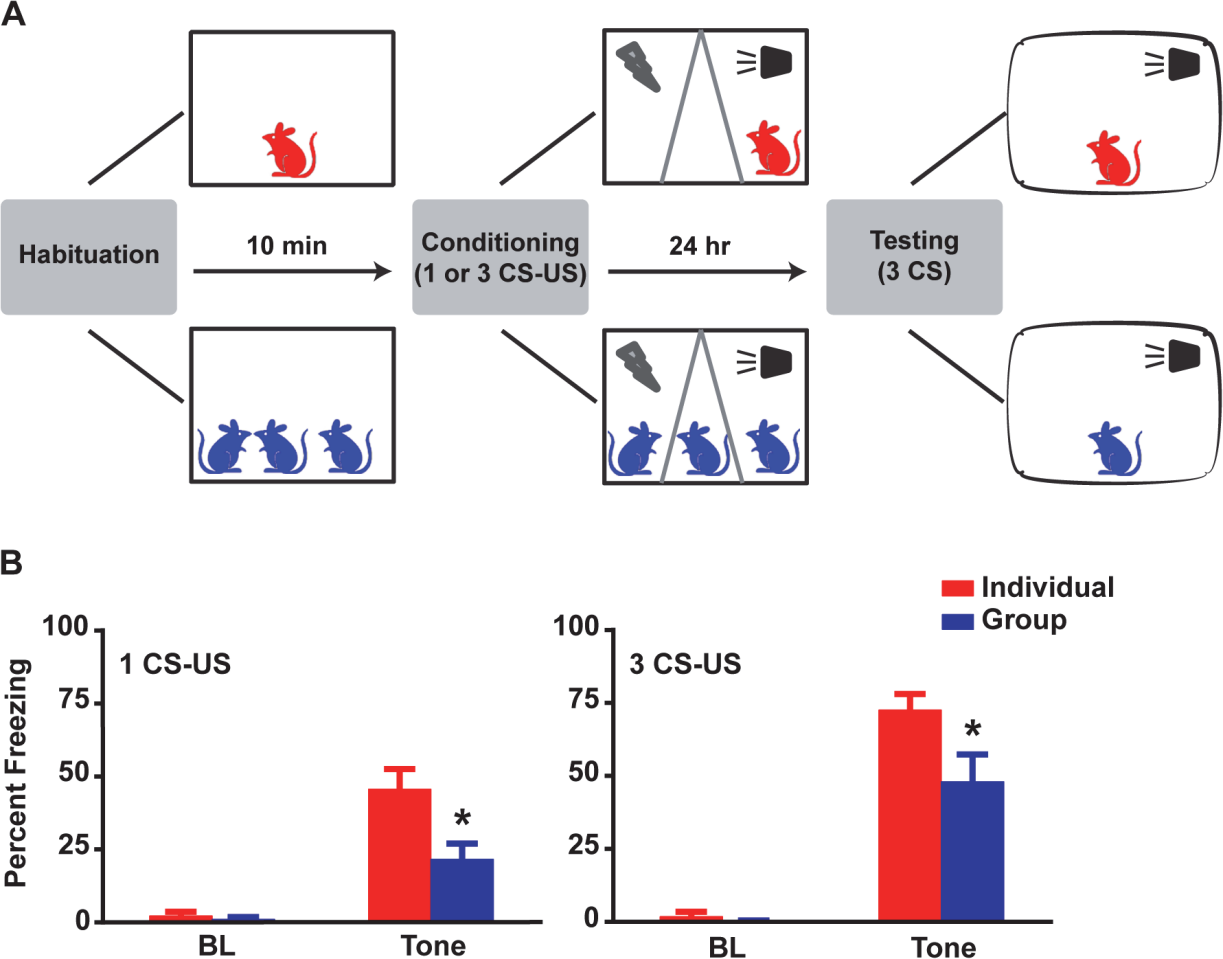
Group conditioning causes a robust reduction in subsequent fear expression. (A) Schematic: rats are divided between Individual and Group conditions. After a 10-min period, a transparent insert is placed and rats are assigned to 1 of 3 compartments. A single or three tones (CS) co-terminating with a footshock (US) are delivered. Twenty-four hours later, the rats are exposed to three CS presentations in a different context for LTM testing. (B) Group rats freeze significantly less than Individuals during CS presentation while maintaining similar low levels of Pre-CS baseline (BL) freezing. Two-way Repeated Measures (RM) ANOVA revealed an effect of condition (Individual or Group), F (1, 34) = 8.93, p<0.01, and an interaction (BL x CS [average of 3]), F (1, 34) = 6.12, p<0.05. Post-hoc tests revealed no differences for BL, p>0.1, and a robust reduction in Group CS freezing, p<0.001. This was replicated in a LTM test for 3 CS-US deliveries during conditioning. Two-way RM ANOVA revealed an effect of condition, F (1, 31) = 5.05, p<0.05, and an interaction, F (1, 31) = 4.17, p<0.05. Post-hoc tests revealed no differences for BL, p>0.1, and a significant reduction in Group CS freezing, p<0.01. Data presented as mean + sem.

We tested whether an aversive event experienced in a group as opposed to an individual setting modified an expected fearful behavioral outcome. To do so, we developed a paradigm for simultaneous fear conditioning of a group of rats (Fig 1A). Animals were housed in triads for three weeks before the start of an experiment. On day 1, rats were allowed to habituate for 10 minutes in the conditioning context alone (Individual, n = 18 rats) or with their cagemates (Group, n = 18 rats). Immediately prior to CS delivery, a custom-made transparent Plexiglas insert was placed in the chamber and Group rats were randomly assigned to one of three compartments while the Individual rat was randomly placed in the left, center or right compartment. A tone (CS) that co-terminated with a mild electric foot-shock (US) was immediately presented. Twenty-four hours later, all rats were individually exposed to three non-reinforced CS presentations in a different context to test for long-term memory (LTM) and their freezing behavior was measured using automated Video Freeze system [11]. Group conditioning resulted in a marked reduction in freezing as compared to Individual conditioning (Figs 1B and S1). On average, Group rats spent approximately 22% of CS exposure time freezing whereas Individual rats spent approximately 46% (p<0.01, ANOVA). This robust reduction of fear was replicated with a stronger conditioning protocol using 3 CS-US pairings in a different cohort of rats (Figs 1B and S1). On average, Group rats spent approximately 48% of CS exposure time freezing whereas Individual rats spent approximately 73% (p<0.05, ANOVA). Thus, experience of fear in a group setting leads to a reliable and robust decrease in fear expression as measured by freezing behavior.

**Fig 2:**
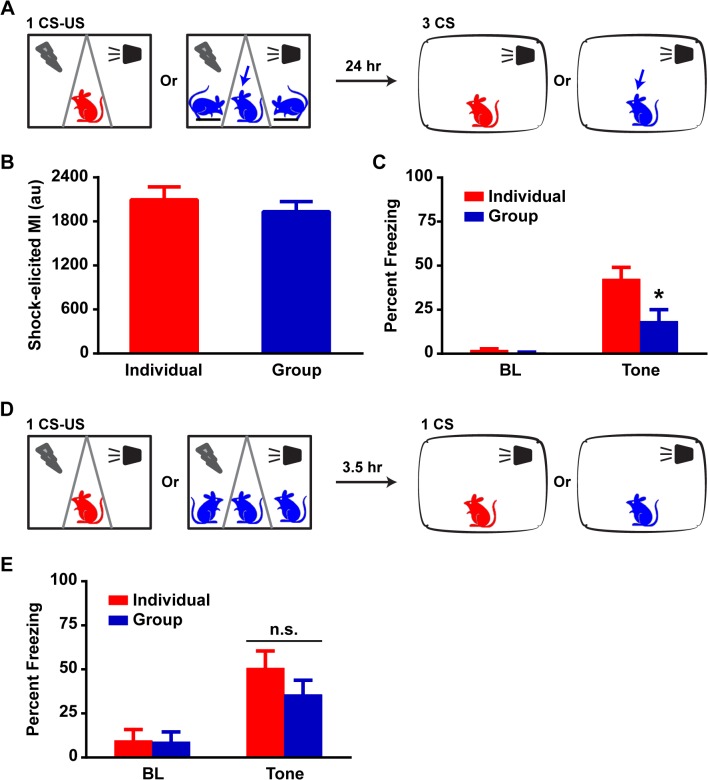
Group-induced fear reduction occurs despite similar fear acquisition in Individuals and Groups. (A) Schematic: in each Group conditioning context, 2 rats are anesthetized and placed on platforms while the third rat is placed awake in the center compartment. Twenty-four hours later, Individual and non-anesthetized Group rats are exposed to three CS presentations in a different context for LTM testing. (B) Shock-induced MI revealed no significant differences between Individuals and Groups (unpaired t-test, p>0.1). (C) Group rats freeze significantly less than Individuals during CS presentation. Two-way RM ANOVA revealed an effect of condition, F (1, 31) = 7.00, p<0.05, and an interaction, F (1, 31) = 5.90, p<0.05. Post-hoc tests revealed no differences for BL, p>0.1, and a significant reduction in Group CS freezing, p<0.01. (D) Schematic: Individuals and Groups are fear-conditioned with 1 CS-US pair and are exposed 3.5 hours later to a single CS presentation for STM testing. (E) Individuals and Groups displayed similar levels of freezing during CS presentation. Two-way RM ANOVA did not reveal any significant effect of condition, F (1, 28) = 0.85, p>0.1 or an interaction, F (1, 28) = 1.25, p>0.1. MI: motion index; au: arbitrary unit. Data presented as mean + sem.

To characterize the potential mechanisms that mediate this fear reduction in Group rats, we first assessed whether animals in a Group had a differential experience of the actual shock delivery. The measurement of shock-induced motion can be used as a surrogate marker of potential differences in the rats’ physical experience of the aversive stimulus and thus the magnitude of fear acquisition. The automated system can accurately and reliably measure individual rat motion during shock delivery [11]; however, it is necessary to limit movement inside the chamber to one rat even as all three receive the shock. Thus, rats underwent fear conditioning as described above with the following modification. Two out of the three Group rats were anesthetized and placed on platforms inside two compartments such that only their feet touched the grid floors while the third rat was placed awake in the third compartment (Fig 2A). Thus, only one average motion index per chamber could be generated (Fig 2B). Analysis of the non-anesthetized rats’ reaction to shock revealed that Group rats (n = 18) experienced the aversive stimulus similarly to Individual rats (n = 15) suggesting that the reduction in freezing is not secondary to differences in shock intensity (p>0.05, ANOVA). Interestingly, awake Group rats spent approximately 18% of CS exposure time freezing whereas Individual rats spent approximately 42% (p<0.05, ANOVA) when tested for fear expression twenty-four hours following conditioning (Fig 2C). The replication of the Group effect under these experimental conditions suggests that direct observation of a conspecific’s reaction to stress cannot explain the fear reduction, as the anesthetized rats clearly did not transmit any perceptible signals of distress. Rather, it appears that the concurrent presence of cagemates during shock delivery, or the group experience of fear, is the component mitigating the subsequent fearful response.

Measurement of short-term memory (STM) is a common method for the assessment of fear acquisition since it is conducted prior to the time frame when the majority of the molecular machinery underlying memory consolidation occurs [13]. We thus conducted a STM test in a separate cohort of rats approximately 3.5 hours after fear conditioning (Fig 2D). There were no significant differences between Group (n = 15) and Individual (n = 15) rats (Fig 2E) for CS exposure time spent freezing (p>0.1, ANOVA). These data argue strongly that fear acquisition was similar in both groups and that neuronal mechanisms mediating fear memory consolidation and/or expression are involved in the markedly attenuated fear memory in Group animals.

**Fig 3:**
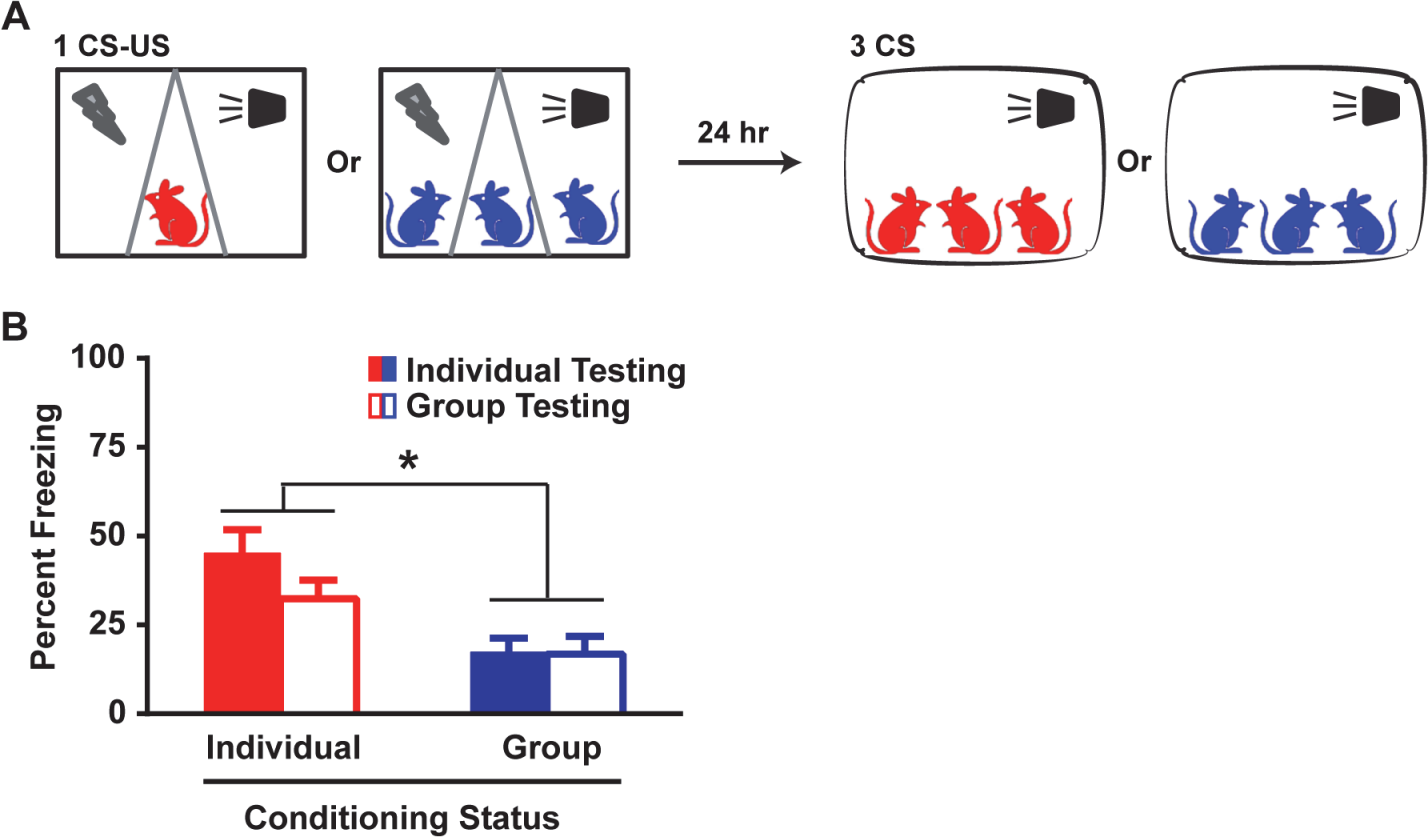
Group-induced fear reduction is dependent on the co-presence of cagemates during conditioning but not testing. (A) Schematic for group testing. Individual and Group rats are fear-conditioned with 1 CS-US pair as described. Twenty-four hours later, the rats are exposed to a LTM test in group with their cagemates and their freezing scores are determined manually. (B) Individual and Group rats maintained respective similar levels of freezing during a LTM test whether they were tested individually or in group. Two-way ANOVA revealed an effect of conditioning (Individual or Group), F (1, 68) = 16.89, p<0.001, but not testing (individually or in group), F (1, 68) = 1.63, p>0.1, or an interaction (conditioning x testing), F (1, 68) = 1.34, p>0.1. Data presented as mean + sem.

Fear reduction in Group rats could however be secondary to the absence, during testing, of cagemates whose presence is potentially critical to the expression of the fear memory. Group (n = 18) and Individual (n = 18) rats were now tested for fear LTM in a group with their cagemates (Fig 3A). Manual scoring of freezing behavior, separately validated against automated scoring (S2 Fig), revealed that conditioning but not testing as an Individual or Group caused the subsequent reduction (Conditioning: p<0.001; Testing: p>0.1, ANOVA) in fear expression (Fig 3B and S1 and S2 Videos). Thus, the Group effect was independent of the presence or absence of cagemates during fear memory testing.

**Fig 4:**
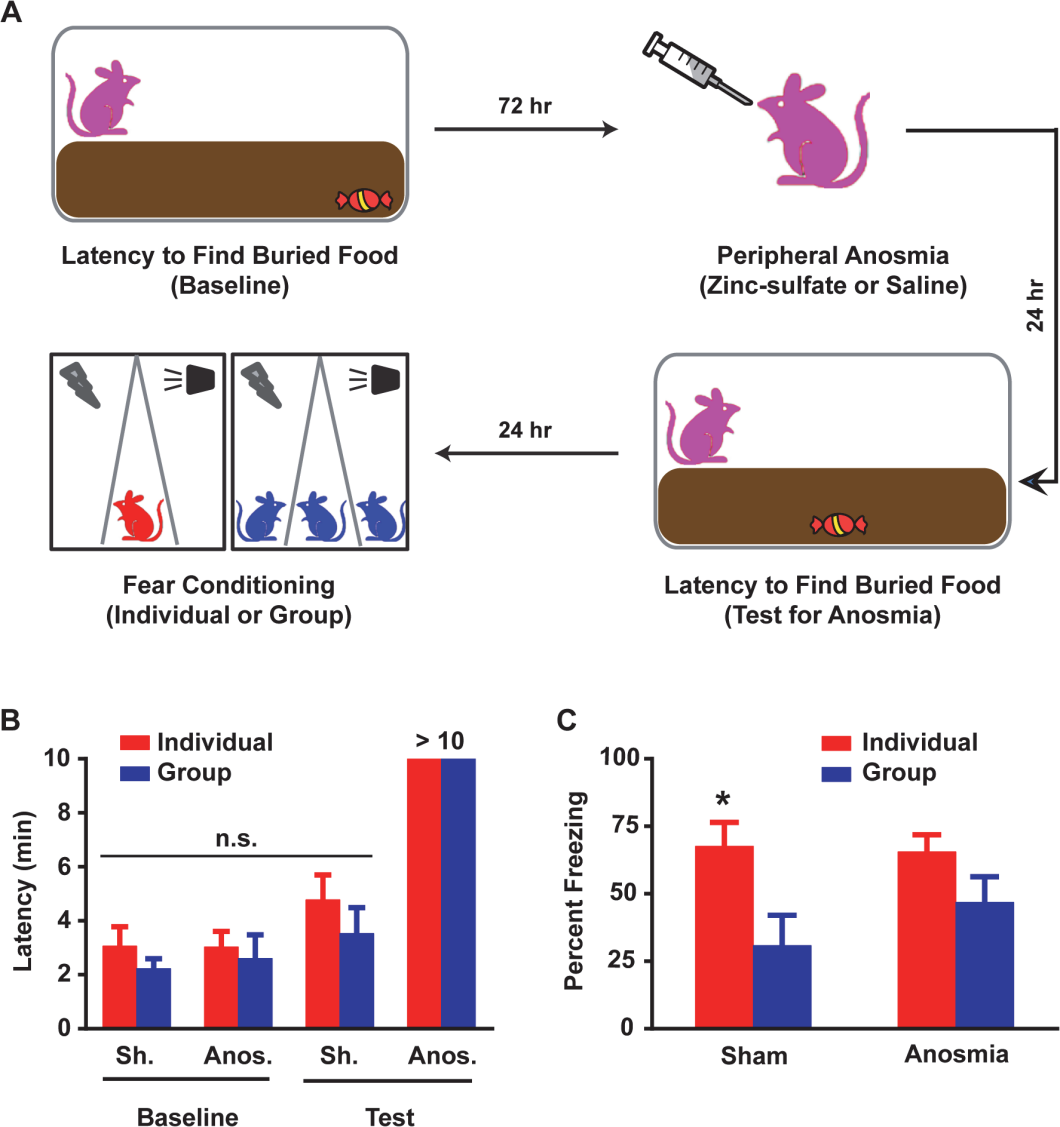
Olfactory signals among Group rats play a significant role in mediating conditioned fear reduction. (A) Schematic for zinc sulfate-induced peripheral anosmia. Rats are subjected to a buried food test. Seventy-two hours later, the rats undergo zinc-sulfate induced peripheral anosmia or sham procedure and the buried food test is repeated the following day to ensure intervention success. Twenty-four hours later, the rats are randomly assigned to Individual or Group conditioning. (B) Zinc sulfate induced peripheral anosmia that was characterized by >10 min latency on the buried food test in both Individual and Group rats. All rats displayed similar baseline pre-treatment latencies which were also similar to sham Individual and Group rat latencies; two way ANOVA, p>0.1. (C) Group rats with peripheral anosmia displayed freezing levels similar to those in Individual anosmic rats while sham Group rats maintained the fear reduction in comparison to sham Individual rats in a LTM test. Two-way ANOVA revealed an effect of conditioning (Individual or Group), F (1, 29) = 9.28, p<0.01. Post-hoc tests revealed that this difference was exclusively driven by the sham rats (Individual vs Group), p<0.05, but not anosmic rats (Individual vs Group), p>0.1. Data presented as mean + sem.

The anesthetized rats experiment suggested that Group rats’ detection of the concurrent presence of their cagemates during fear conditioning was critical for subsequent fear memory reduction. We focused our investigation on olfaction as a highly pertinent sensory modality in rodents and because olfactory signals from experimentally naïve conspecifics have been shown to independently mitigate fear responses in conditioned rats [14]. We therefore subjected both Individual and Group rats to zinc sulfate induced peripheral anosmia or sham exposure [12] prior to fear conditioning (Fig 4A). A buried food test was used to ascertain that the four different groups [Sham Individual (7), Anosmia Individual, (9), Sham Group (8) and Anosmia Group (9)] were matched for olfaction at baseline and to verify the efficacy of the manipulation. Prior to drug or sham exposure, all rats were able to find the treat in approximately 3 minutes and there were no significant differences among the different assigned conditions (Fig 4B). Following exposure, sham rats were able to find the treat in approximately 4 minutes, which was not significantly different from baseline, whereas anosmic rats were not able to find the treat by the cutoff limit of 10 minutes (Fig 4B). Twenty-four hours later, the rats underwent fear conditioning as described (Fig 1A). As expected, sham exposure had no effect on Group-induced fear reduction as Group rats spent approximately 31% of CS exposure time freezing, significantly less (p<0.05, ANOVA *post hoc*) than Individual rats at approximately 68% (Fig 4C). On the other hand, anosmia increased freezing levels during CS exposure in Group rats to approximately 47%, which was not significantly different (p>0.1, ANOVA *post hoc*) from that of Individual rats at 66% (Fig 4C). The effect of anosmia on Group fear conditioning was replicated subsequently with the stronger conditioning protocol (S3 Fig). Thus, anosmia appears to render Group rats’ fear memory more similar to that of Individuals.

## Discussion

Our study demonstrates that the experience of fear in a group causes a marked reduction in the expression of that particular fear memory. Considerable interest in rodent emotional contagion, in general [15–17], and fear transmittance [18–23], in particular, has begun to re-emerge recently. These studies examine the structure of associative learning between experimentally naïve animals and conspecifics that undergo fearful experiences. Interestingly, and depending on the particular experimental setup, fear learning through social observation has led both to enhanced [18,19,22] and attenuated [20,24] fearful behavior in observer rodents exposed to fear-conditioned demonstrators. Our study differs from this literature by asking an equally relevant social and biological question; namely, how an aversive experience undertaken in a group of animals modulates subsequent fear memory. As our experiment with anesthetized rats clearly demonstrates, learning specifically through conspecifics’ distress cannot account for our data. Together, our shock-induced motion reactivity and short-term memory experiments, argue that alterations in acquisition of fear learning, in general, also did not contribute to the Group fear memory.

The expression of fear reduction is not contingent on the group experience at both conditioning and testing phases of the experiment. In other words, rats need not experience fearful reminders also in a group setting for the reduction in fearful behavior to be manifest. Put differently, if the group effect is to be perceived as “protective”, it is also enduring beyond the initial confines of the fearful experience. This is important clinically as victims of collective trauma or stress invariably experience their posttraumatic symptoms individually. These symptoms have been associated with, among others, an inability to inhibit fear [25,26]. Thus, fear conditioning models, while not completely representative, can help better understand the neurobiology of PTSD [10]. Understanding the neural mechanisms mediating fear reduction following an aversive event experienced in a group will complement the significant body of research on heritable genetic profiles [27,28] addressing the critical question of susceptible versus resilient phenotype emergence following a traumatic experience.

Taken together, similar fear acquisition between Individual and Group rats, the persistence of the Group effect even when some group members are anesthetized, and the lack of effect of *testing* as Individual or Group, suggest that fear reduction is a consequence of altered memory consolidation of the collective experience of an aversive event in a group. A considerable portion of this group “awareness” seems to be mediated through olfaction as Group rats subjected to anosmia freeze at similar levels to Individual rats while sham Group rats freeze significantly less. In rodents, olfactory and vomeronasal inputs to the medial amygdala have been shown to play a role in the detection of conspecifics [29]. Importantly, ample evidence exists that the medial amygdala and its hypothalamic projections constitute relay stations in the neural circuits of fear of aggressive conspecifics and fear of predators, which are quite distinct from the well-studied circuitry of learned fear [30]. Thus, specific olfactory inputs to the amygdala can potentially modulate distinct efferent pathways and their respective outputs [31]. Our Group paradigm may tap into aspects of emotional memory that more closely model conscious fear in humans. A better understanding of the neural mechanisms underlying Group fear circuits and how they interact in ethologically relevant models will provide new insight into the complex human pathologies of anxiety and fear.

## Supporting Information

S1 Fig Group fear conditioning causes a robust reduction in subsequent expression of cue-elicited fear across tone presentations. Right, two-way Repeated Measures (RM) ANOVA revealed an effect of condition (Individual or Group), F (1, 34) = 8.10, p<0.01, an effect of CS presentation (BL, T1, T2, T3), F (3, 102) = 29.01, p<0.0001, and an interaction (condition x CS presentation), F (3, 102) = 3.11, p<0.05. Post-hoc tests revealed no differences for BL, p>0.1, near significant reduction for T1, p = 0.07 and a significant reductions for both T2 and T3, p<0.01 and <0.05, respectively. Left, the finding was replicated in a LTM test for 3 CS-US presentations during conditioning. Two-way Repeated Measures (RM) ANOVA revealed an effect of condition (Individual or Group), F (1, 31) = 4.83, p<0.05, an effect of CS presentation (BL, T1, T2, T3), F (3, 93) = 69.00, p<0.0001, and a near significant interaction (condition x CS presentation), F (3, 93) = 2.69, p = 0.05. Post-hoc tests revealed no differences for BL or T1, p>0.1, near significant reduction for T2, p = 0.07 and a significant reduction for T3, p<0.05. Data presented as mean + sem.
(TIF)

S2 Fig Linear fit and correlation for manual versus automated scoring reveal ideal parameters. Group testing necessitated manual scoring for accurate determination of freezing levels of each rat. Despite high correlation and fit (n = 36 rats), we manually re-scored individually-tested rats for a more rigorous analysis depicted in Fig 3.
(TIF)

S3 Fig Olfactory signals among Group rats play a significant role in mediating fear reduction following a strong conditioning paradigm. Right, zinc sulfate-induced peripheral anosmia that was characterized by >10 min latency on the buried food test in both Individual and Group rats. All rats displayed similar baseline pre-treatment latencies which were also similar to Individual and Group rat latencies after sham treatment; two way ANOVA, p>0.1. Left, Group rats with peripheral anosmia displayed freezing levels similar to those in Individual anosmic rats while sham Group rats maintained the fear reduction in comparison to sham Individual rats in a LTM test (performed 4 days after conditioning). Two-way ANOVA revealed an effect of conditioning (Individual or Group), F (1, 20) = 7.70, p<0.05. Post-hoc tests revealed that this difference was exclusively driven by the sham rats (Individual vs Group), p<0.05, but not anosmic rats (Individual vs Group), p>0.1. Data presented as mean + sem.
(TIF)

S1 Video Individual rats tested in a group. Representative video of group testing of rats that were fear-conditioned as Individuals. Beep indicates the start of the 30-sec CS delivery. The presence of more than one rat in the testing chamber does preclude the significant freezing behavior displayed or the ability of a blinded experimenter to score it. Individual rats maintain significant freezing during CS delivery.
(MP4)

S2 Video Group rats tested in a group. Representative video of group testing of rats that were fear-conditioned in a Group. Beep indicates the start of the 30-sec CS delivery. The presence of more all the cagemates that were shocked together 24 hours earlier does not increase the freezing behavior displayed by each rat. Group rats maintain their low freezing scores during CS delivery.
(MP4)

## References

[1] Mahan AL, Ressler KJ. Fear conditioning, synaptic plasticity and the amygdala: implications for posttraumatic stress disorder. Trends Neurosci. 2012;35(1):24–35. 10.1016/j.tins.2011.06.007 21798604 PMC3206195

[2] Rothbaum BO, Davis M. Applying learning principles to the treatment of post-trauma reactions. Ann N Y Acad Sci. 2003;1008:112–21. 14998877 10.1196/annals.1301.012

[3] Blair HT, Schafe GE, Bauer EP, Rodrigues SM, LeDoux JE. Synaptic plasticity in the lateral amygdala: a cellular hypothesis of fear conditioning. Learn Mem. 2001;8(5):229–42. 11584069 10.1101/lm.30901

[4] Kim JJ, Jung MW. Neural circuits and mechanisms involved in Pavlovian fear conditioning: a critical review. Neurosci Biobehav Rev. 2006;30(2):188–202. 16120461 10.1016/j.neubiorev.2005.06.005 PMC4342048

[5] Maren S, Quirk GJ. Neuronal signalling of fear memory. Nat Rev Neurosci. 2004;5(11):844–52. 15496862 10.1038/nrn1535

[6] Myers KM, Davis M. Mechanisms of fear extinction. Mol Psychiatry. 2007;12(2):120–50. 17160066 10.1038/sj.mp.4001939

[7] Pape H-C, Pare D. Plastic synaptic networks of the amygdala for the acquisition, expression, and extinction of conditioned fear. Physiol Rev. 2010;90(2):419–63. 10.1152/physrev.00037.2009 20393190 PMC2856122

[8] Bouton ME, Bolles RC. Conditioned fear assessed by freezing and by the suppression of three different baselines. Anim Learn Behav. 1980;8(3):429–34.

[9] Santiago PN, Ursano RJ, Gray CL, Pynoos RS, Spiegel D, Lewis-Fernandez R, et al A systematic review of PTSD prevalence and trajectories in DSM-5 defined trauma exposed populations: intentional and non-intentional traumatic events. PLOS One. 2013;8(4):e59236 10.1371/journal.pone.0059236 23593134 PMC3623968

[10] Yehuda R, LeDoux J. Response variation following trauma: a translational neuroscience approach to understanding PTSD. Neuron. 2007;56(1):19–32. 17920012 10.1016/j.neuron.2007.09.006

[11] Anagnostaras SG, Wood SC, Shuman T, Cai DJ, Leduc AD, Zurn KR, et al Automated assessment of pavlovian conditioned freezing and shock reactivity in mice using the video freeze system. Front Behav Neurosci. 2010;4:1–11. 10.3389/neuro.08.001.2010 20953248 PMC2955491

[12] Masini C V, Garcia RJ, Sasse SK, Nyhuis TJ, Day HEW, Campeau S. Accessory and main olfactory systems influences on predator odor-induced behavioral and endocrine stress responses in rats. Behav Brain Res. 2010;207(1):70–7. 10.1016/j.bbr.2009.09.038 19800371 PMC2787960

[13] Johansen JP, Cain CK, Ostroff LE, LeDoux JE. Molecular Mechanisms of Fear Learning and Memory. Cell. Elsevier Inc.; 2011;147(3):509–24. 10.1016/j.cell.2011.10.009 22036561 PMC3215943

[14] Kiyokawa Y, Honda A, Takeuchi Y, Mori Y. A familiar conspecific is more effective than an unfamiliar conspecific for social buffering of conditioned fear responses in male rats. Behav Brain Res. 2014;267:189–93 10.1016/j.bbr.2014.03.043 24698797

[15] Langford DJ, Tuttle AH, Brown K, Deschenes S, Fischer DB, Mutso A, et al Social approach to pain in laboratory mice. Soc Neurosci. 2010;5(2):163–70. 10.1080/17470910903216609 19844845

[16] Ben-Ami Bartal I, Decety J, Mason P. Empathy and pro-social behavior in rats. Science. 2011;334(6061):1427–30. 10.1126/science.1210789 22158823 PMC3760221

[17] Prehn-Kristensen A, Wiesner C, Bergmann TO, Wolff S, Jansen O, Mehdorn HM, et al Induction of empathy by the smell of anxiety. PLOS One. 2009;4(6):e5987 10.1371/journal.pone.0005987 19551135 PMC2695008

[18] Knapska E, Mikosz M, Werka T, Maren S. Social modulation of learning in rats. Learn Mem. 2010;17(1):35–42. 10.1101/lm.1670910 20042480 PMC3960044

[19] Bruchey AK, Jones CE, Monfils M-H. Fear conditioning by-proxy: social transmission of fear during memory retrieval. Behav Brain Res. Elsevier B.V.; 2010;214(1):80–4. 10.1016/j.bbr.2010.04.047 20441779 PMC2975564

[20] Bredy TW, Barad M. Social modulation of associative fear learning by pheromone communication. Learn Mem. 2009;16(1):12–8. 10.1101/lm.1226009 19117912 PMC2632855

[21] Sanders J, Mayford M, Jeste D. Empathic fear responses in mice are triggered by recognition of a shared experience. PLOS One. 2013;8(9):e74609 10.1371/journal.pone.0074609 24058601 PMC3776853

[22] Jeon D, Kim S, Chetana M, Jo D, Ruley HE, Lin S-Y, et al Observational fear learning involves affective pain system and Cav1.2 Ca2+ channels in ACC. Nat Neurosci. Nature Publishing Group; 2010;13(4):482–8. 10.1038/nn.2504 20190743 PMC2958925

[23] Zhou Y, Won J, Karlsson MG, Zhou M, Rogerson T, Balaji J, et al CREB regulates excitability and the allocation of memory to subsets of neurons in the amygdala. Nat Neurosci. 2009;12(11):1438–43. 10.1038/nn.2405 19783993 PMC2783698

[24] Guzmán YF, Tronson NC, Guedea A, Huh KH, Gao C, Radulovic J. Social modeling of conditioned fear in mice by non-fearful conspecifics. Behav Brain Res. 2009;201(1):173–8. 10.1016/j.bbr.2009.02.024 19428631 PMC2680762

[25] Jovanovic T, Norrholm SD, Fennell JE, Keyes M, Fiallos AM, Myers KM, et al Posttraumatic stress disorder may be associated with impaired fear inhibition: relation to symptom severity. Psychiatry Res. 2009;167(1–2):151–60. 10.1016/j.psychres.2008.02.003 19345420 PMC2713500

[26] Milad MR, Orr SP, Lasko NB, Chang Y, Rauch SL, Pitman RK. Presence and acquired origin of reduced recall for fear extinction in PTSD: results of a twin study. J Psychiatr Res. 2008;42(7):515–20. 10.1016/j.jpsychires.2008.01.017 18313695 PMC2377011

[27] Mahan AL, Ressler KJ. Fear conditioning, synaptic plasticity and the amygdala: implications for posttraumatic stress disorder. Trends Neurosci. 2012;35(1):24–35. 10.1016/j.tins.2011.06.007 21798604 PMC3206195

[28] Jovanovic T, Ressler KJ. How the neurocircuitry and genetics of fear inhibition may inform our understanding of PTSD. Am J Psychiatry. 2010;167(6):648–62. 10.1176/appi.ajp.2009.09071074 20231322 PMC3603297

[29] Kollack-Walker S, Don C, Watson SJ, Akil H. Differential expression of c-fos mRNA within neurocircuits of male hamsters exposed to acute or chronic defeat. J Neuroendocrinol. 1999;11(7):547–59. 10444312 10.1046/j.1365-2826.1999.00354.x

[30] Gross CT, Canteras NS. The many paths to fear. Nat Rev Neurosci. Nature Publishing Group; 2012;13(9):651–8. 10.1038/nrn3301 22850830

[31] Motta SC, Goto M, Gouveia FV, Baldo MVC, Canteras NS, Swanson LW. Dissecting the brain’s fear system reveals the hypothalamus is critical for responding in subordinate conspecific intruders. Proc Natl Acad Sci U S A. 2009;106(12):4870–5. 10.1073/pnas.0900939106 19273843 PMC2660765

