# Correction: The Fragrant Power of Collective Fear

**DOI:** 10.1371/journal.pone.0131149

**Published:** 2015-06-18

**Authors:** The PLOS ONE Staff

The last author’s name is spelled incorrectly. The correct name is: Jane R. Taylor. The correct citation is: Harb R, Taylor JR (2015) The Fragrant Power of Collective Fear. PLoS ONE 10(5): e0123908. doi:10.1371/journal.pone.0123908. The publisher apologizes for the error.
