# Supplementary figures and images for: The Fragrant Power of Collective Fear
Source: PLoS One. 2015 May 6;10(5):e0123908. doi: 10.1371/journal.pone.0123908 (PMC4422730; doi:10.1371/journal.pone.0123908)

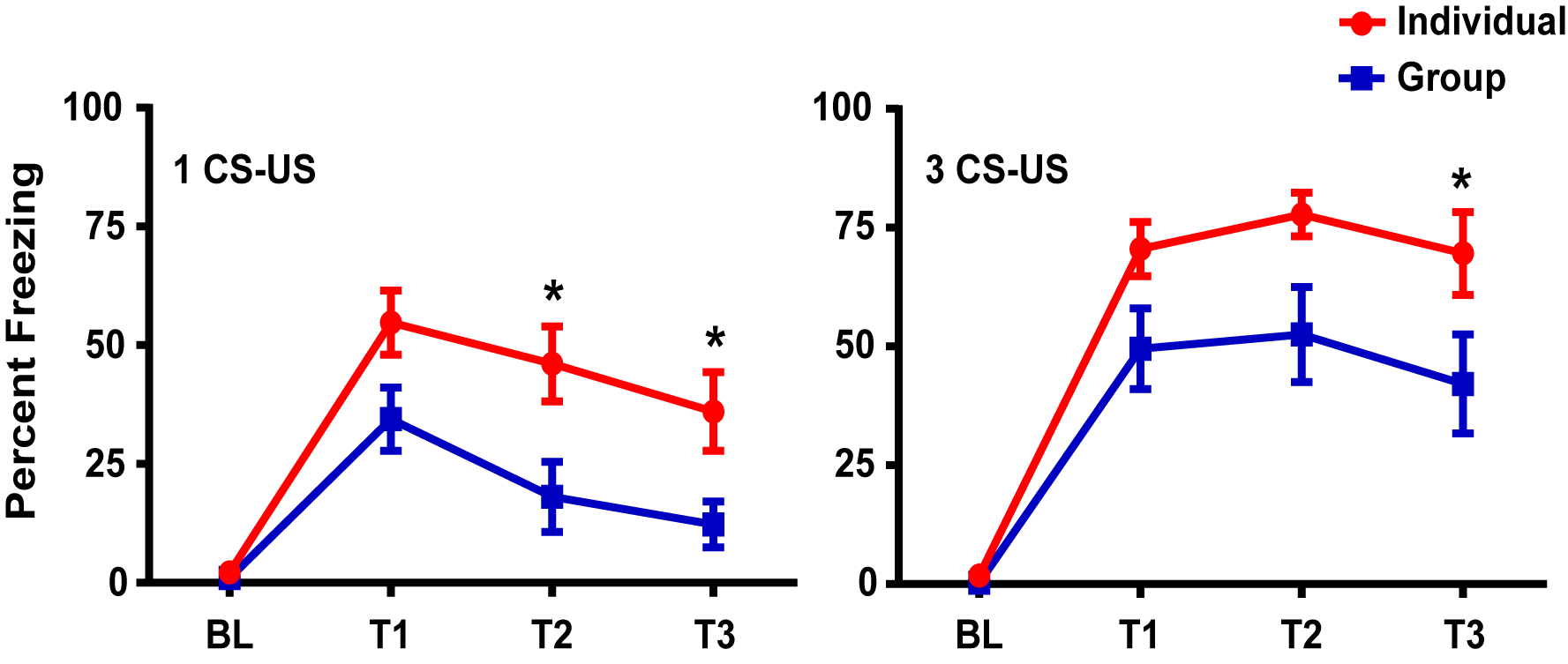

Supplement: S1 Fig — Right, two-way Repeated Measures (RM) ANOVA revealed an effect of condition (Individual or Group), F (1, 34) = 8.10, p<0.01, an effect of CS presentation (BL, T1, T2, T3), F (3, 102) = 29.01, p<0.0001, and an interaction (condition x CS presentation), F (3, 102) = 3.11, p<0.05. Post-hoc tests revealed no differences for BL, p>0.1, near significant reduction for T1, p = 0.07 and a significant reductions for both T2 and T3, p<0.01 and <0.05, respectively. Left, the finding was replicated in a LTM test for 3 CS-US presentations during conditioning. Two-way Repeated Measures (RM) ANOVA revealed an effect of condition (Individual or Group), F (1, 31) = 4.83, p<0.05, an effect of CS presentation (BL, T1, T2, T3), F (3, 93) = 69.00, p<0.0001, and a near significant interaction (condition x CS presentation), F (3, 93) = 2.69, p = 0.05. Post-hoc tests revealed no differences for BL or T1, p>0.1, near significant reduction for T2, p = 0.07 and a significant reduction for T3, p<0.05. Data presented as mean + sem. (TIF) [file pone.0123908.s001.tif]

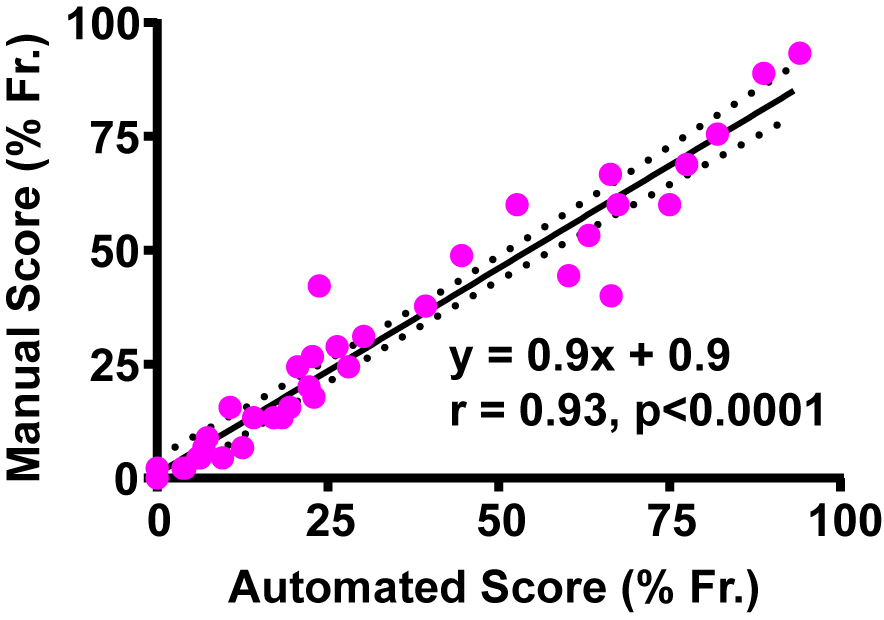

Supplement: S2 Fig — Group testing necessitated manual scoring for accurate determination of freezing levels of each rat. Despite high correlation and fit (n = 36 rats), we manually re-scored individually-tested rats for a more rigorous analysis depicted in Fig 3. (TIF) [file pone.0123908.s002.tif]

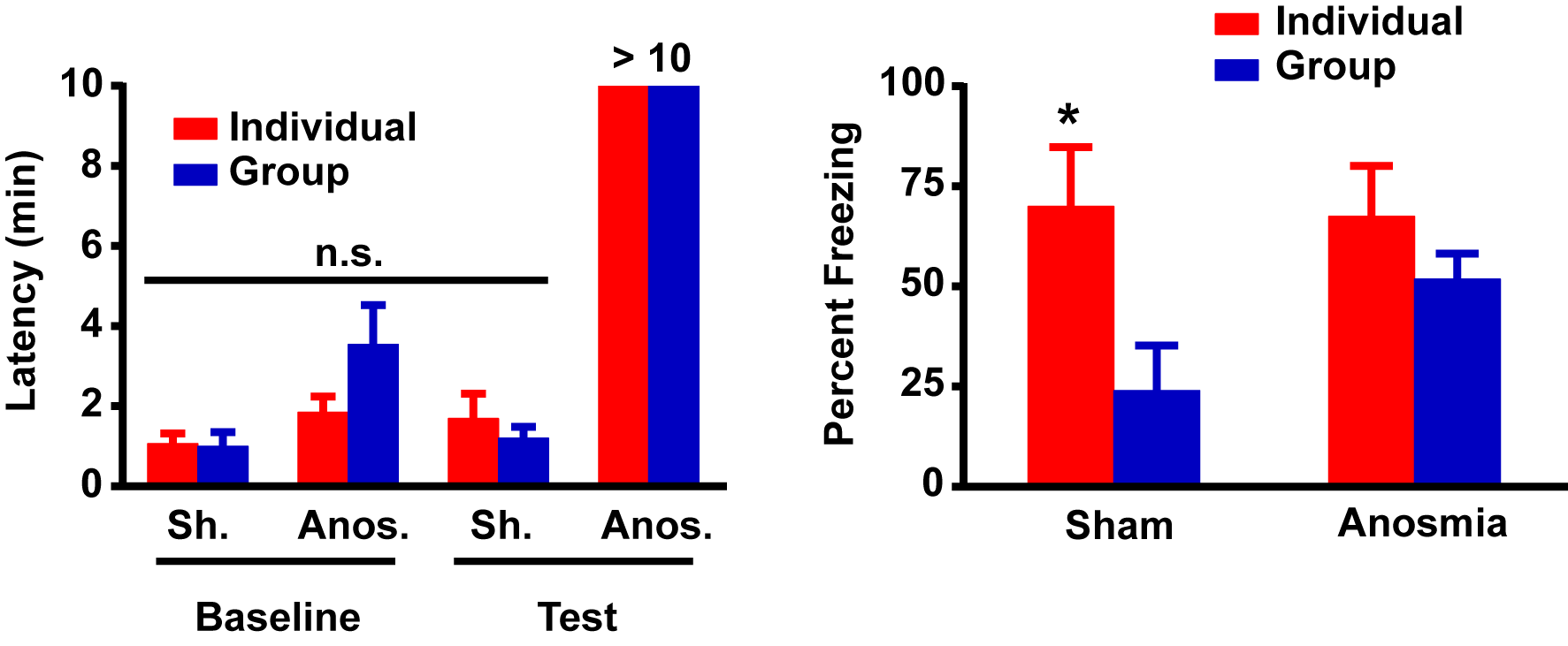

Supplement: S3 Fig — Right, zinc sulfate-induced peripheral anosmia that was characterized by >10 min latency on the buried food test in both Individual and Group rats. All rats displayed similar baseline pre-treatment latencies which were also similar to Individual and Group rat latencies after sham treatment; two way ANOVA, p>0.1. Left, Group rats with peripheral anosmia displayed freezing levels similar to those in Individual anosmic rats while sham Group rats maintained the fear reduction in comparison to sham Individual rats in a LTM test (performed 4 days after conditioning). Two-way ANOVA revealed an effect of conditioning (Individual or Group), F (1, 20) = 7.70, p<0.05. Post-hoc tests revealed that this difference was exclusively driven by the sham rats (Individual vs Group), p<0.05, but not anosmic rats (Individual vs Group), p>0.1. Data presented as mean + sem. (TIF) [file pone.0123908.s003.tif]
